# Is Single-Dose Antimicrobial Prophylaxis Sufficient to Control Infections in Gastrointestinal Oncological Surgeries?

**DOI:** 10.7759/cureus.16939

**Published:** 2021-08-06

**Authors:** Amudhan Kannan, Mirunalini Ravichandran, Sudharsanan Sundaramurthi, Myat Win, Anjli Tara, Sheila W Ruo, Waleed Sultan, Vijaya Lakshmi Yanamala, Abdul Rub Hakim Mohammed, Jerry Lorren Dominic

**Affiliations:** 1 General Surgery, California Institute of Behavioral Neurosciences & Psychology, Fairfield, USA; 2 Medicine, Jawaharlal Institute of Postgraduate Medical Education and Research, Puducherry, IND; 3 Pharmacology, Jawaharlal Institute of Postgraduate Medical Education and Research, Puducherry, IND; 4 General Surgery, Jawaharlal Institute of Postgraduate Medical Education and Research, Puducherry, IND; 5 General Surgery, Nottingham University Hospitals NHS Trust, Nottingham, GBR; 6 General Surgery, Liaquat University of Medical and Health Sciences, Jamshoro, PAK; 7 Faculty of Medicine, Beni Suef University, Beni Suef, EGY; 8 Neurology, California Institute of Behavioral Neurosciences & Psychology, Fairfield, USA; 9 Surgery, Halifax Health Medical Center, Daytona Beach, USA; 10 Emergency Medicine, Ronald Reagan Institute of Emergency Medicine, Washington, USA; 11 Medicine, Vinayaka Mission's Kirupananda Variyar Medical College, Salem, IND; 12 General Surgery, Stony Brook Southampton Hospital, New York, USA; 13 General Surgery and Orthopaedic Surgery, Cornerstone Regional Hospital, Edinburg, USA

**Keywords:** surgical site infections, wound infections, antimicrobial prophylaxis, gastrointestinal cancer surgery, esophageal cancer surgery, gastric cancer surgery, colorectal cancer surgery, single-dose antimicrobial prophylaxis

## Abstract

Surgical site infections (SSIs) represent one of the most important complications occurring postoperatively following surgical procedures. The SSI incidence is higher following gastrointestinal (GI) surgeries compared to any other surgery. It contributes to the majority of morbidity and mortality in patients undergoing GI surgeries. The accepted practice worldwide for the prevention and control of SSIs is providing antimicrobial prophylaxis. The appropriate antimicrobial and dose are chosen depending on the microbial flora, complications, and patient risk factors. The objective of this review was to determine the sufficient number of prophylactic antimicrobial doses that would be efficacious and safe in controlling the SSIs following GI oncological surgeries. Single-dose antimicrobial prophylaxis has shown the same efficacy as the multiple-dose antimicrobial regimen in controlling SSIs in esophageal, gastric, and colorectal surgeries. The advantages of a single-dose regimen include less chance of emergence of resistance, less chance for allergies or toxicity, and less cost. The addition of metronidazole with single-dose antimicrobial prophylaxis in colorectal surgery should be considered due to its beneficial effect in further reducing infections. Further randomized controlled trials are needed for the literature to determine the efficacy and safety of single-dose antimicrobial prophylaxis in patients undergoing esophageal and colorectal surgeries. In addition, studies are required to determine the individual effectiveness of metronidazole in controlling SSIs in colorectal surgeries.

## Introduction and background

**Figure 1:**
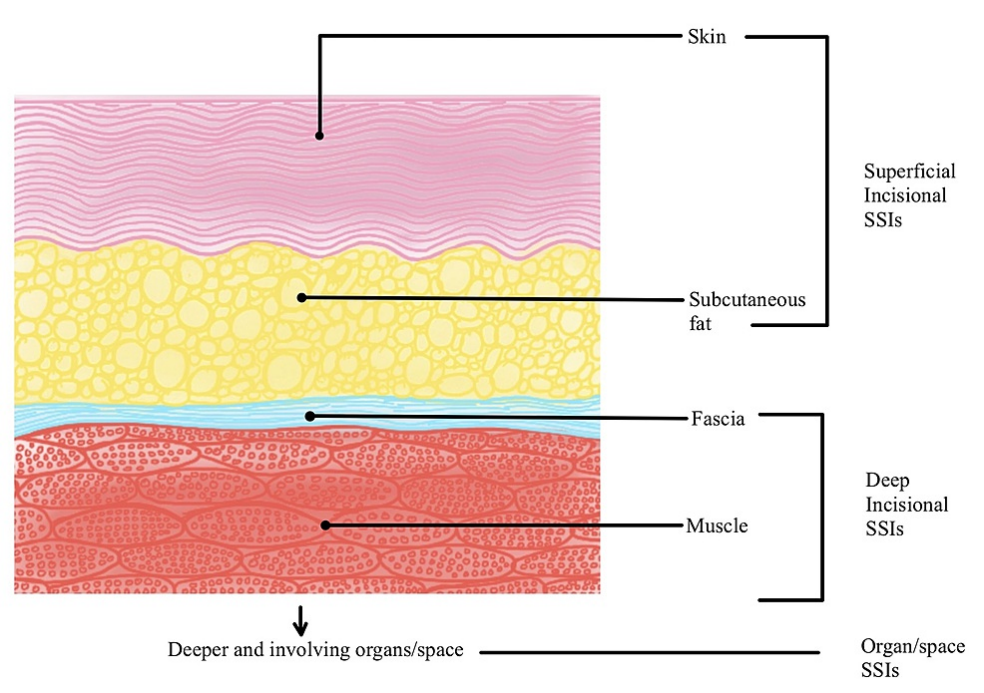
Types of SSIs based on the depth of involvement SSIs, surgical site infections

In any surgery, there is always a process of breaching the normal protective barriers of the human body. There is an increased risk of infections when protective barriers such as skin and mucous membrane are cut through for manipulation. Surgical site infections (SSIs) represent one of the most important complications occurring postoperatively following surgeries [1]. SSIs can be classified into types based on the depth of the infection. It can involve the skin and subcutaneous fat (superficial incisional SSIs), deep tissues such as muscles (deep incisional SSIs), or even extending beyond these limits (organ/space SSIs). SSIs further complicate the postoperative outcomes in patients who undergo surgery, thus negatively impacting the health and wellness of the patient. It not only contributes to morbidity and mortality but also to the cost and quality of life. Therefore, it is crucial to prevent and control SSIs. The accepted practice worldwide for preventing and controlling SSIs is providing antimicrobial prophylaxis to patients undergoing any surgical procedures. The types of SSIs are shown in Figure 1.

Out of the surgical procedures, gastrointestinal (GI) surgeries pose an important unique issue in terms of SSIs. Patients undergoing GI surgeries have a high risk of SSIs following elective or emergency GI operations than other surgeries. This is because the GI tract serves as a home for many microbial florae that have the potential to breach the mucosal barrier during the surgery and spread superficially or to other places to cause infections. Thus, the incidence of SSIs is higher following GI surgeries than any other surgery, with an average incidence being 10%-25% reported in various studies [2,3]. Surgical procedures in the GI tract can involve the esophagus, stomach, small intestine, colon, and rectum. This list can also include biliary surgeries. One of the most common indications for GI surgery is cancer.

For SSI antimicrobial prophylaxis, the antimicrobial drugs can be given either as a single-dose regimen or multiple-dose regimen in which the postoperative continuation of antimicrobials is warranted. However, each regimen has its strengths and limitations, which vary among different surgical procedures. There are reviews published in the literature comparing the efficacy of a single-dose regimen to multiple-dose antimicrobial prophylaxis in preventing and controlling SSIs in patients undergoing orthognathic procedures, orthopedic procedures, and other major surgeries. In the review article by McDonald et al., the authors concluded that there was no clear superiority of either single dose or more extended duration antimicrobial prophylaxis in preventing infection in major surgeries [4]. Marcussen et al. reported that a single dose of preoperative antimicrobial decreased infection and alveolar osteitis in lower third molar surgical extraction applying osteotomy [5]. In the meta-analysis by Slobogean et al. comparing single- versus multiple-dose antibiotic prophylaxis in preventing SSIs in the surgical treatment of closed fractures, the results could not demonstrate the superiority of multiple-dose antimicrobial prophylaxis over a single-dose regimen [6].

Single-dose antimicrobial prophylaxis is more economical and medically desirable [7]. However, there is a high risk of the emergence of resistance as antimicrobials are given for a longer duration in the case of multiple-dose antimicrobial prophylaxis. In addition to contributing to the emergence of microbial resistance, antimicrobial therapy for a more extended period increases the risk of allergic reactions and adverse events in those patients [7]. Despite the proven efficacy, safety, and importance of antimicrobial prophylaxis in GI surgeries, there is no clear explanation for the sufficient number of doses of antimicrobial therapy that is efficacious and safe to control and prevent SSIs in GI surgeries. Thus, the purpose of this traditional review is to study the effectiveness and safety of single-dose antimicrobial prophylaxis compared to multiple-dose antimicrobial prophylaxis in the control and prevention of SSIs in patients undergoing GI oncological surgeries.

## Review

Method

This traditional review was designed, and its results were reported using the Scale for the Assessment of Narrative Review Articles (SANRA) guidelines and checklist [8].

We included predominantly randomized controlled trials (RCTs) to provide the highest level of evidence with minimal bias and errors. This study did not have any constraint on any age limit. The studies published between January 2005 and May 2021 were included. Among the studies chosen, it was confirmed that all the studies included human subjects and published in the English language. Studies that did not have full-text available were excluded. Databases used to retrieve articles included PubMed and Cochrane library. These databases were examined for pertinent articles that could be included in this review using appropriate keywords; the keywords and Medical Subject Headings (MeSH) terms used included “Anti-Bacterial Agents,” “Stomach neoplasms,” “Colorectal neoplasms,” “Esophageal neoplasms,” “Surgical wound infections,” “Single-dose antibiotic prophylaxis,” and “Multiple-dose antibiotic prophylaxis.” The Boolean search method was used in PubMed to combine the keywords and MeSH words. Two authors (AK and MR) independently searched PubMed and Cochrane library using the keywords mentioned above and selected the relevant articles based on titles and abstract. Full-text articles of the selected studies were retrieved, and three authors (AK, MR, and SS) examined all the full-text articles for eligibility and relevance. Other authors helped in the final assessment of the included studies. The quality assessment of the included RCTs was performed using the Cochrane Handbook for Systematic Reviews of Interventions. One of the authors (JLD) evaluated the risk of bias in the chosen RCTs.

Results

The preliminary database searches identified a total of 47 articles. Of these, 14 articles that were found to be duplicates were subsequently removed. In PubMed, we applied filters for study type, studies involving humans, and studies in the English language. In the Cochrane library, in addition to year, we applied the filters including study type as trials, and source as Embase. After the application of the filters, a total of 15 articles were identified. We did the preliminary screening of these articles by reading the titles and abstracts, and we excluded seven articles that were not relevant and did not meet the inclusion criteria.

**Figure 2:**
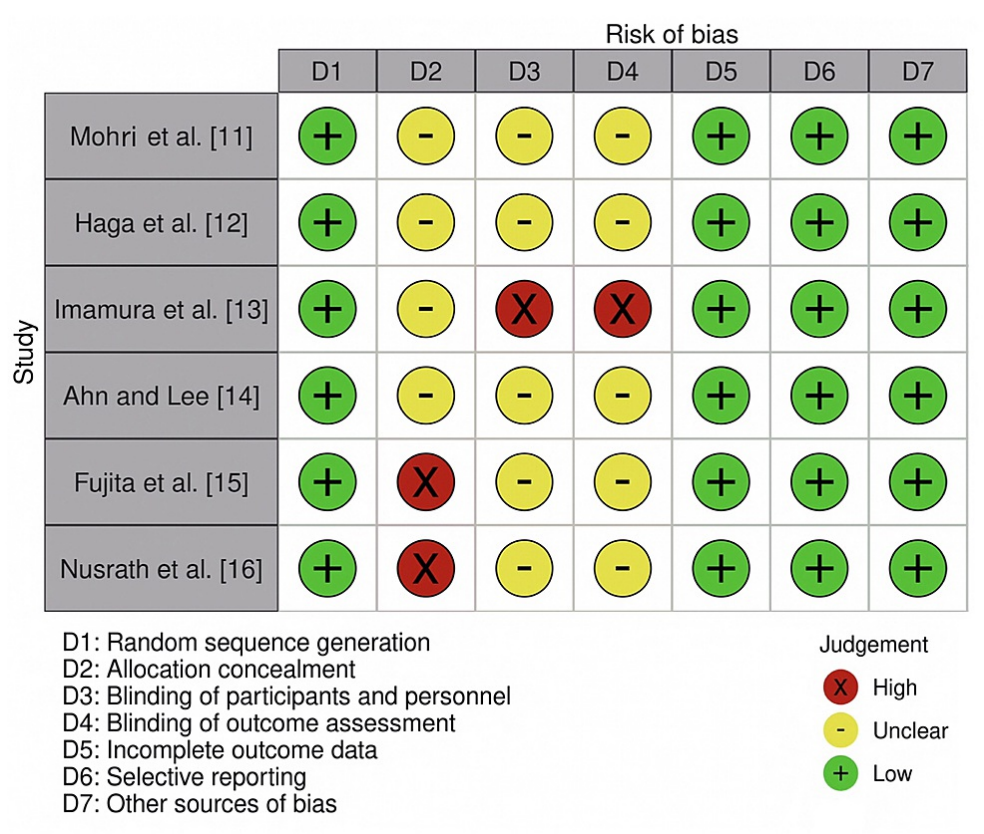
Risk of bias in the included randomized controlled trials D, domain The randomized controlled trials included for the risk of bias assessment are studies by Mohri et al. [11], Haga et al. [12], Imamura et al. [13], Ahn and Lee [14], Fujita et al. [15], and Nusrath et al. [16].

**Figure 3:**
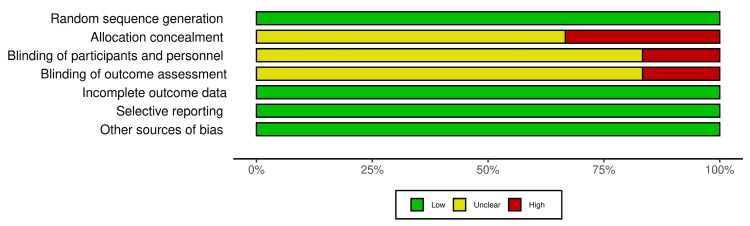
Summary of risk of bias of all the included randomized controlled trials

A total of eight studies were included for the review. Of the eight studies, two studies included patients who underwent esophageal cancer surgery. Three studies were on patients who underwent gastric cancer surgeries, and three studies were on patients who underwent colorectal cancer surgeries. Six out of the eight studies were RCTs, and no RCTs involved esophageal cancer surgery. The two studies on esophageal cancer were non-comparative prospective and retrospective studies. The study characteristics are presented in Table 1. The risk of bias assessment and the summary of the risk of bias of all the included RCTs are shown in Figures 2, 3.

**Table 1:** Characteristics of the included studies in this review RCT, randomized controlled trial; SD, single-dose group; MD, multiple-dose group

Study	Type of study	Type of cancer	Sample size	Details of the surgical procedure	Mean duration of operation in minutes (SD vs MD)
Ruol et al. (2000) [9]	Prospective non-comparative study	Esophageal cancer	82	Total esophageal resection = 3	Median operative length = 350 minutes
				Partial esophageal resection = 79	
Hochreiter et al. (2018) [10]	Retrospective study	Esophageal cancer	173	Transthoracic esophagectomy with abdominal and mediastinal lymphadenectomy for all the included patients	275 vs 262.1 (not significant)
Mohri et al. (2007) [11]	RCT	Gastric cancer	486	Total/proximal gastrectomy = 172 (SD = 78; MD = 94)	232 vs 234 (not significant)
				Distal gastrectomy = 288 (SD = 147; MD = 141)	
				Wedge resection = 3 (SD = 2; MD = 1)	
				Gastrojejunostomy = 23 (SD = 16; MD = 7)	
Haga et al. (2012) [12]	RCT	Gastric cancer	325	Total gastrectomy = 132 (SD = 66; MD = 66)	181.5 vs 185 (not significant)
				Proximal / distal gastrectomy = 193 (SD = 98; MD = 95)	
Imamura et al. (2012) [13]	RCT	Gastric cancer	355	Distal gastrectomy plus lymphadenectomy for all the included patients	209 vs 200
Ahn and Lee (2012) [14]	RCT	Colorectal cancer	93	Low anterior resection = 38 (SD = 20; MD = 18)	207 vs 212 (not significant)
				Right hemicolectomy = 23 (SD = 10, MD = 13)	
				Left hemicolectomy = 6 (SD = 5, MD = 1)	
				Other types = 26 ( SD = 13; MD = 13)	
Fujita et al. (2007) [15]	RCT	Colorectal cancer	377	Conventional method = 262 (SD = 129; MD = 133)	178.8 vs 170 (not significant)
				Laparoscopic method = 115 (SD = 61; MD = 54)	
Nusrath et al. (2020) [16]	RCT	All clean-contaminated oncological surgeries	315	Open = 120 (SD = 45; MD = 75)	Not mentioned
				Laparoscopic = 69 (SD = 44; MD = 25)	
				Laparoscopic assisted = 126 (SD = 70; MD = 56)	

Discussion

Esophageal Oncological Surgeries: Appropriate Dose for Controlling SSIs and Other Infectious Complications

Esophageal cancer though rare is an invasive and aggressive disease with a poor prognosis and very low survival rate. The poor prognosis is due to various factors such as very late presentation of disease and other surgical procedural difficulties [9,17]. The treatment for esophageal cancer is resection of the tumor, which provides positive outcomes and improvement in the quality of life [9]. The infectious postoperative complications of esophageal surgery include SSIs, abscesses, pneumonia, and urinary tract infections. The serious complications include anastomotic leak and fistula. One of the leading causes of complication in the early postoperative days of esophageal surgery is pneumonia. Compared to other GI surgeries, esophageal surgeries are usually associated with a longer duration of operation, more blood loss, and a different microflora in the operative field [10]. Esophageal surgeries involve both the thorax and abdomen. Staphylococci and gram-negative bacteria are the causative organisms of SSIs and abscesses. But polymicrobial aerobic and anaerobic organisms are also commonly detected [18].

There are not many published studies in the literature that compared the efficacy of single-dose antimicrobial prophylaxis to multiple-dose antimicrobial prophylaxis in esophageal surgeries. In Ruol et al.'s prospective non-comparative study, a total of 82 patients with esophageal cancer who underwent surgery were included. These patients were given a single dose of ceftriaxone as antimicrobial prophylaxis. In addition, three doses of metronidazole were given (preoperatively and postoperatively). Only one patient had postoperative SSI though the overall infection rate was 17%. Even though this study was a non-comparative prospective study, the results showed that a single dose of antimicrobial agent plus metronidazole could provide adequate prophylaxis and less costly for patients undergoing major esophageal surgeries [9].

In another retrospective study by Hochreiter et al., 173 patients who underwent esophageal resection were studied to determine whether multiple-dose antimicrobial prophylaxis helps in reducing postoperative pneumonia and improve mortality. Out of 173 patients,104 patients received only a single dose of moxifloxacin (control group). The remaining patients received a five-day postoperative therapy with moxifloxacin. In addition, Mezlocillin and metronidazole were given to all patients in both groups. SSI was seen in one patient in the multiple-dose group and two patients in the single-dose group. It showed no statistically significant difference between the two groups [10].

To the best of our knowledge, there are no RCTs in the literature on patients who underwent esophageal cancer surgery. In the studies mentioned above, i.e., Ruol et al. and Hochreiter et al., the single-dose antimicrobial prophylaxis was as effective as the multiple-dose regimen in controlling the SSIs and other infections [9,10]. Metronidazole was added along with the prophylactic antimicrobial agent in both studies. The exact role of the addition of metronidazole has to be further studied in patients undergoing esophageal surgery. The characteristics of the studies included and the incidence of SSIs are summarized in Table 2.

**Table 2:** Study characteristics and incidence of SSIs in esophageal oncological surgery SSI, surgical site infection

Study	Year published	Type of study	Single-dose group (n)	Multiple-dose group (n)	SSI in the single-dose group	SSI in the multiple-dose group
Ruol et al. [9]	2000	Non-comparative prospective study	82	No multi-dose group	1 (1.2%)	Not applicable
Hochreiter et al. [10]	2018	Retrospective study	104	69	2 (1.9%)	1 (1.4%)

Efficacy and Safety of Single-Dose Versus Multiple-Dose Regimen in Gastric Surgeries for Gastric Cancer

Though not prevalent in western countries, gastric cancer is one of the most commonly diagnosed cancers in Asian countries like China, Japan, and India [19,20]. The commonly done gastric surgeries include distal gastrectomy, total gastrectomy, and sub-total gastrectomy. These procedures involve multiple intestinal anastomosis and manipulation of the liver and pancreas, leading to fistula formation and subsequent infection. Thus, gastric surgeries are associated with a higher incidence of SSIs. According to the studies published in the literature, the incidence of SSI following gastric surgeries is approximately 10%, and thus, the prevention of SSIs is crucial, like in other surgeries, in gastric surgeries [21]. One of the studies in the literature that studied the risk factors of SSIs after gastrectomy is the study by Migita et al. In this study, 842 patients who underwent gastrectomy were studied. The predictors of organ/space SSI were duration of operation, male gender, corticosteroid therapy, and total gastrectomy. The duration of hospital stay and re-operation rates were higher in patients who developed SSIs [19]. As gastric cancer is prevalent in Asian countries, it was found in various studies that the use of antimicrobial prophylaxis for the longer duration in gastric surgeries was widespread in Asian countries. Even though the importance of antimicrobial prophylaxis is well-known and accepted, the ideal number of doses of antimicrobial prophylaxis to prevent SSIs following gastric surgeries remains ambiguous.

In this review, we managed to retrieve only a few RCTs to provide the highest level of evidence. These RCTs included patients with gastric cancer who underwent gastric surgeries to determine the effectiveness of single-dose antimicrobial prophylaxis in controlling SSIs. In this RCT by Mohri et al., patients with gastric cancer were randomized into two groups: single-dose prophylaxis group (n = 243) and multi-dose prophylaxis group (n = 243). The antibiotics used were cefazolin or ampicillin-sulbactam. The difference in the SSI incidence between the two groups was 0.9% (9.5% in the single-dose group vs. 8.6% in the multi-dose group). The incidence of SSIs did not show a significant difference between the two groups. Moreover, on subgroup analysis of patients receiving either cefazolin or ampicillin-sulbactam combination, there was no significant difference between the two antibiotic subgroups in terms of incidence of SSI [11]. A very similar result was reported by the randomized study by Haga et al. In this study, though the overall incidence of SSIs was less in the multiple-dose group, there was no statistically significant difference between the single-dose and the multiple-dose groups [12]. Imamura et al. conducted an RCT to study the incidence of SSIs with intraoperative antimicrobial prophylaxis and intraoperative plus postoperative administration in a total of 355 patients who underwent distal gastrectomy for gastric cancer. In the intraoperative group (n = 176), 5% patients had SSIs compared to 9% in the extended group (n = 179). The statistical analysis showed that a single dose of intraoperative antimicrobial prophylaxis (which was given before the surgical incision and every three hourly as intraoperative supplements) was non-inferior to the multiple-dose prophylaxis [13].

All the studies mentioned above reported that single-dose antimicrobial prophylaxis for gastric cancer surgery was as effective as multiple-dose antimicrobial prophylaxis for controlling SSIs in patients undergoing gastric cancer surgery. The use of a multiple-dose antimicrobial regimen in gastric surgeries was not recommended in any of the studies, as mentioned earlier. The characteristics of the studies included and the incidence of SSIs are summarized in Table 3.

**Table 3:** Study characteristics and incidence of SSIs in gastric oncological surgery SSI, surgical site infection; RCT, randomized controlled trial

Study	Year published	Type of study	Single-dose group (n)	Multiple-dose group (n)	SSI in the single-dose group	SSI in the multiple-dose group
Mohri et al. [11]	2007	RCT	243	243	23 (9.5%)	21 (8.6%)
Haga et al. [12]	2012	RCT	164	161	15 (9.1%)	10 (6.2%)
Imamura et al. [13]	2012	RCT	176	179	8 (4.5%)	16 (8.9%)

Colorectal Oncological Surgery: The Role of Antimicrobial Prophylaxis and Addition of Metronidazole to the Regimen

SSIs are ubiquitous in colorectal surgeries compared to gastric surgeries and other GI surgeries [22,23]. Surgical site infections have been associated with an increased duration of hospital stay, higher readmission rates, morbidity, and mortality. According to the published data, colorectal procedures are associated with 10%-15% of all SSIs [24-26]. The increased incidence of SSIs in colorectal surgeries is due to the presence of a vast majority of bacterial flora in the colon, especially in the distal colon. Colorectal cancer by itself has a very poor survival rate. In addition to the complications of colorectal surgery, SSIs contribute to the decrease in quality of life by increasing the period of hospital stay, readmissions, and other complications. Thus, antimicrobial prophylaxis plays a vital role in the prevention of SSI in patients undergoing colorectal surgeries.

There are not many published RCTs comparing the efficacy of single-dose antimicrobial prophylaxis in patients who underwent colorectal surgeries. One of the RCTs by Ahn and Lee included patients who underwent elective colorectal surgery. A total of 93 patients were included and were assigned to either the single-dose group (n = 48) or the three-dose group (n = 45). The antibiotic given was a second-generation cephalosporin and metronidazole. The overall postoperative infection rate did not differ between the two groups. The SSI incidence did not show any significant difference between the groups (11.1% in multiple-dose vs 10.4% in single-dose). Therefore, the number of doses was not found as an independent risk factor for SSIs. The crucial point to consider in this RCT is the addition of metronidazole along with the first-line agent in both groups [14].

Metronidazole plays a critical role in the prevention and reduction of SSIs, especially in colorectal surgeries. The importance of the addition of metronidazole is shown in the meta-analysis by Nelson et al. This meta-analysis reported that additional coverage aerobic and anaerobic organisms both showed statistically significant improvements in SSIs [27]. The common sources of microbial contamination of the surgical site in colorectal surgery are colonic flora, small intestinal flora, and skin flora. The organisms include aerobes and anaerobes. Metronidazole helps in controlling SSIs by covering anaerobes. Fujita et al. conducted an RCT in Japan to study the efficacy of single-dose antimicrobial prophylaxis in colorectal surgery. A total of 384 patients were randomized to either the single-dose group or the three-dose group. In this study, metronidazole was not given to both groups. Analysis of incisional SSIs showed that incisional SSI was significantly lower in the three-dose group (4.3%) than the single-dose group (14.2%). The multi-variate analysis also showed a significant association of single-dose antibiotics with an increased risk of incisional SSI. On analysing organ or space SSI incidence, there was no significant difference between the two groups. The authors concluded that single-dose antibiotic prophylaxis is not efficacious in colorectal surgeries unless combined with metronidazole [15].

In the RCT by Nusrath et al., 105 patients who had colorectal malignancy were included. The incidence of SSI was seen more in the group that received an extended dose of antibiotics compared to the single-dose group. In addition, this study reported a higher incidence of remote infection in the single-dose group. However, the analysis and results showed that the single-dose antimicrobial prophylaxis was as efficacious as the multiple-dose regimen in controlling the SSIs [16].

Thus, single-dose antimicrobial prophylaxis with metronidazole may be considered an effective regimen in controlling the SSIs in patients undergoing colorectal surgeries. However, in addition to increased cost and increased risk of adverse events, the multiple-dose antibiotic regimen is associated with increased *Clostridium difficile* infection, which was proved by the meta-analysis by Nelson et al. [28]. Another Cochrane meta-analysis by Nelson et al. also reported that though SSIs were marginally higher with single‐dose antibiotics, the regimen can be considered as benefits are more [27]. The characteristics of the studies included and the incidence of SSIs are summarized in Table 4.

**Table 4:** Study characteristics and incidence of SSIs in colorectal oncological surgery SSI, surgical site infection; RCT, randomized controlled trial

Study	Published year	Type of study	Single-dose group (n)	Multiple-dose group (n)	SSI in the single-dose group	SSI in the multiple-dose group
Ahn and Lee [14]	2013	RCT	48	45	5 (10.4%)	5 (11.1%)
Fujita et al. [15]	2007	RCT	190	187	40 (21.1%)	24 (12.8%)
Nusrath et al. [16]	2020	RCT	53	52	10 (18.9%)	13 (25%)

Limitations

This review included RCTs predominantly due to the highest level of evidence they provide with only a few errors. Due to the scarcity of RCTs and other types of studies on this topic, only a limited number of studies were found appropriate to be included for this review. As most of the studies included in this review were randomized clinical trials, the sample size in some of the studies was limited. Only oncological GI surgeries were studied due to the availability of comprehensive data.

## Conclusions

The objective of this review was to determine the sufficient number of prophylactic antimicrobial doses that would be efficacious and safe in controlling the SSIs in GI surgeries. SSIs contribute to the majority of morbidity and mortality in patients undergoing GI surgeries. In addition, they contribute to increased duration of hospital stay, thereby indirectly increasing the total cost incurred to a patient. Using antimicrobials for a more extended period in GI surgeries contributes to the development of resistance in microbes and allergies, and the total cost. The appropriate antimicrobial and dose are chosen depending on the microbial flora, complications, and patient risk factors.

Single-dose antimicrobial prophylaxis has shown the same efficacy as the multiple-dose antimicrobial regimen in controlling and preventing SSIs in esophageal, gastric, and colorectal surgeries. A single-dose antimicrobial regimen has the following advantages: less chance of emergence of resistance, less chance for allergies or toxicity, and less cost. Thus, in patients undergoing GI surgeries, single-dose antimicrobial prophylaxis would be sufficient to control major SSIs and complications. The addition of metronidazole to single-dose antimicrobial prophylaxis, especially in colorectal surgery, should be considered due to its beneficial effect in further reducing SSIs. We recommend further randomized controlled trials to determine the efficacy and safety of single-dose antimicrobial prophylaxis in patients undergoing esophageal and colorectal surgeries to provide high-quality evidence with minimal bias. In addition, further studies are needed to determine the individual efficacy of metronidazole in controlling the SSIs in colorectal surgeries.

## References

[1] Chodak GW, Plaut ME (1977). Use of systemic antibiotics for prophylaxis in surgery: a critical review. Arch Surg.

[2] Aga E, Keinan-Boker L, Eithan A, Mais T, Rabinovich A, Nassar F (2015). Surgical site infections after abdominal surgery: incidence and risk factors. A prospective cohort study. Infect Dis (Lond).

[3] Legesse Laloto T, Hiko Gemeda D, Abdella SH (2017). Incidence and predictors of surgical site infection in Ethiopia: prospective cohort. BMC Infect Dis.

[4] McDonald M, Grabsch E, Marshall C, Forbes A (1998). Single- versus multiple-dose antimicrobial prophylaxis for major surgery: a systematic review. Aust N Z J Surg.

[5] Marcussen KB, Laulund AS, Jørgensen HL, Pinholt EM (2016). A systematic review on effect of single-dose preoperative antibiotics at surgical osteotomy extraction of lower third molars. J Oral Maxillofac Surg.

[6] Slobogean GP, Kennedy SA, Davidson D, O'Brien PJ (2008). Single- versus multiple-dose antibiotic prophylaxis in the surgical treatment of closed fractures: a meta-analysis. J Orthop Trauma.

[7] Childs SJ, deBessonet DA, Merlin AS (1984). Antibiotic prophylaxis in elective genitourinary tract surgery: a comparison of single-dose pre-operative cefotaxime and multiple-dose cefoxitin. J Antimicrob Chemother.

[8] Baethge C, Goldbeck-Wood S, Mertens S (2019). SANRA—a scale for the quality assessment of narrative review articles. Res Integr Peer Rev.

[9] Ruol A, Bertiato G, Boscarin S (2000). Short-term prophylaxis with ceftriaxone plus metronidazole in esophageal cancer surgery. J Chemother.

[10] Hochreiter M, Uhling M, Sisic L (2018). Prolonged antibiotic prophylaxis after thoracoabdominal esophagectomy does not reduce the risk of pneumonia in the first 30 days: a retrospective before-and-after analysis. Infection.

[11] Mohri Y, Tonouchi H, Kobayashi M, Nakai K, Kusunoki M (2007). Randomized clinical trial of single- versus multiple-dose antimicrobial prophylaxis in gastric cancer surgery. Br J Surg.

[12] Haga N, Ishida H, Ishiguro T, Kumamoto K, Ishibashi K, Tsuji Y, Miyazaki T (2012). A prospective randomized study to assess the optimal duration of intravenous antimicrobial prophylaxis in elective gastric cancer surgery. Int Surg.

[13] Imamura H, Kurokawa Y, Tsujinaka T (2012). Intraoperative versus extended antimicrobial prophylaxis after gastric cancer surgery: a phase 3, open-label, randomised controlled, non-inferiority trial. Lancet Infect Dis.

[14] Ahn BK, Lee KH (2013). Single-dose antibiotic prophylaxis is effective enough in colorectal surgery. ANZ J Surg.

[15] Fujita S, Saito N, Yamada T (2007). Randomized, multicenter trial of antibiotic prophylaxis in elective colorectal surgery: single dose vs 3 doses of a second-generation cephalosporin without metronidazole and oral antibiotics. Arch Surg.

[16] Nusrath S, Nair A, Dasu S (2020). Single-dose prophylactic antibiotic versus extended usage for four days in clean-contaminated oncological surgeries: a randomized clinical trial. Indian J Surg Oncol.

[17] Coia LR, Sauter ER (1994). Esophageal cancer. Curr Probl Cancer.

[18] Finlay IG, Wright PA, Menzies T, McArdle CS (1982). Microbial flora in carcinoma of oesophagus. Thorax.

[19] Migita K, Takayama T, Matsumoto S (2012). Risk factors for surgical site infections after elective gastrectomy. J Gastrointest Surg.

[20] Han JH, Jeong O, Ryu SY, Jung MR, Park YK (2014). Efficacy of single-dose antimicrobial prophylaxis for preventing surgical site infection in radical gastrectomy for gastric carcinoma. J Gastric Cancer.

[21] Imai E, Ueda M, Kanao K, Miyaki K, Kubota T, Kitajima M (2005). Surgical site infection surveillance after open gastrectomy and risk factors for surgical site infection. J Infect Chemother.

[22] Jeong WK, Park JW, Lim SB, Choi HS, Jeong SY (2010). Cefotetan versus conventional triple antibiotic prophylaxis in elective colorectal cancer surgery. J Korean Med Sci.

[23] Fry DE (2008). Preventive systemic antibiotics in colorectal surgery. Surg Infect (Larchmt).

[24] Hedrick TL, Harrigan AM, Sawyer RG, Turrentine FE, Stukenborg GJ, Umapathi BA, Friel CM (2015). Defining surgical site infection in colorectal surgery: an objective analysis using serial photographic documentation. Dis Colon Rectum.

[25] Anthony T, Murray BW, Sum-Ping JT (2011). Evaluating an evidence-based bundle for preventing surgical site infection: a randomized trial. Arch Surg.

[26] Krieger BR, Davis DM, Sanchez JE, Mateka JJ, Nfonsam VN, Frattini JC, Marcet JE (2011). The use of silver nylon in preventing surgical site infections following colon and rectal surgery. Dis Colon Rectum.

[27] Nelson RL, Gladman E, Barbateskovic M (2014). Antimicrobial prophylaxis for colorectal surgery. Cochrane Database Syst Rev.

[28] Nelson RL, Suda KJ, Evans CT (2017). Antibiotic treatment for Clostridium difficile-associated diarrhoea in adults. Cochrane Database Syst Rev.

